# Dietary Glyceryl Monolaurate Supplementation During Pregnancy Enhances Fetal Intrauterine Development and Antioxidant Capacity in Sows via Microbiota Modulation

**DOI:** 10.3390/antiox14070783

**Published:** 2025-06-25

**Authors:** Zhichao Fu, Jun Wang, Yueqi Zhao, Tanyi Deng, Ziwei Ma, Wutai Guan, Xiangfang Zeng, Fang Chen

**Affiliations:** 1 State Key Laboratory of Swine and Poultry Breeding Industry, College of Animal Science, South China Agricultural University, Guangzhou 510642, China; 2543872938@stu.scau.edu.cn (Z.F.); lilang@stu.scau.edu.cn (J.W.); 2695192284@stu.scau.edu.cn (Y.Z.); dengtanyi@stu.scau.edu.cn (T.D.); 15119852861@stu.scan.edu.cn (Z.M.); wtguan@scau.edu.cn (W.G.); 2 Guangdong Laboratory of Modern Agriculture in Lingnan Guangdong Province, Guangzhou 510642, China; 3 State Key Laboratory of Animal Nutrition, Ministry of Agriculture Feed Industry Center, China Agricultural University, Beijing 100193, China; zengxf@cau.edu.cn

**Keywords:** glyceryl monolaurate, gut microbiota, SCFAs, antioxidative, longissimus dorsi muscle, intestinal

## Abstract

This study elucidates the mechanisms underlying the positive effect of glyceryl monolaurate (GML) on fetal intrauterine development via maternal gut-microbiota modulating effects using a sow model. Addition of GML (1000 mg/kg) improved neonatal intestinal conditions (jejunal villus height, VH/CD ratio and tight junctions) and dorsal longissimus muscle (*MyoD*, *MyoG* and *MSTN*) development in the GML-treated group. Furthermore, GML improved maternal gut microbiota composition by enriching short-chain fatty acid (SCFA)-producing bacteria *Lactobacillus* and *Akkermansia*. Meanwhile, SCFA concentrations in sow feces and newborn plasma, as well as their receptors (GPR41/43) in intestine and muscle were upregulated with GML, corresponding with enhanced antioxidative and anti-inflammatory capacity. Further correlation analysis revealed *Akkermansia* and *Lactobacillus* positively correlated with SCFAs, antioxidative indicators, and anti-inflammatory capacity markers. Moreover, GML inhibited the activation of the MAPK/NF-κB inflammatory signaling pathway. In summary, GML enhanced fetal intrauterine development by modulating sow intestinal SCFA-producing bacteria.

## 1. Introduction

The maternal energy metabolism during pregnancy in sows leads to elevated oxidative stress in the uterine environment, restricting fetal growth and development [1], and leaving newborn piglets vulnerable to oxidative stress, which impairs early growth and causes lasting developmental challenges. It may reduce T-AOC, SOD, and other antioxidant protections that serve as the first line of defence [2]. Oxidative stress-induced redox imbalance can trigger systemic inflammation. Notably, SCFAs are major antioxidative metabolites produced by gut microbiota [3]. Recent research has increasingly focused on the crucial role of maternal gut microbiota in influencing both sow health and fetal development during gestation [4]. The gut microbiota has a profound impact on maternal and infant health dynamics [5]. Microbial metabolites from the maternal gut can enter the circulatory system, due to tight junction proteins (ZO-1/occludin) the barrier for nutrient absorption cross the placental barrier, and directly affect the growth processes of the developing fetus [6]. Notably, SCFAs, a class of gut-derived microbial metabolites, possess potent antioxidants and anti-inflammatory properties. They usually contain fewer than six carbon atoms and can be straight-chain or branched-chain. The most abundant short-chain fatty acids are acetic acid (C2), propionic acid (C3) and butyric acid (C4) [7,8]. Evidence suggests that enhancing the population of SCFA-producing bacteria in the sow’s intestinal tract during pregnancy could help improve sow-related production performance [9]. On the other hand, systemic inflammation triggered by lipopolysaccharides (LPS) is associated with increased mortality rates in neonatal piglets [10]. Despite these findings, there remains a lack of research on whether modulating the maternal gut microbiota in sows can effectively reduce fetal oxidative stress and promote optimal intrauterine development for improved piglet health.

GML, a naturally occurring compound found in both breast milk and coconut oil, exhibits significant antimicrobial, anti-inflammatory, and immunoregulatory properties [11]. Upon intestinal hydrolysis, GML releases lauric acid (modulating PPAR-γ signaling) and glycerol (fueling SCFA production) [12]. Studies have shown that this 12-carbon fatty acid derivative offers various benefits for pigs [9], particularly in improving growth performance and other production traits [13], boosting antioxidant defenses, and reducing inflammatory processes [14]. Research further demonstrates that GML administration induces notable morphological changes in pig intestinal architecture [15], while concurrently alleviating inflammation, enhancing resistance to oxidative stress and modulating immune function [15,16]. In recent years, a large body of literature has reported that adding GML to the diet can effectively improve the gut microbiota structure of animals, including chickens, piglets, and other animals, increasing the abundance of certain beneficial microorganisms and thereby improving gut health [17,18]. Our previous research has found that dietary regulation during pregnancy can alter the gut microbiota structure of sows, thereby changing the abundance of corresponding metabolic products, which leads to alterations in the metabolic products entering the uterus and ultimately affects fetal development [19]. However, it remains unknown whether adding GML to the diet of pregnant sows can improve their gut microbiota and subsequently influence fetal development.

In this study, we evaluated the effect of adding 1000 mg/kg GML on fecal microbiota composition and levels of SCFAs in sows and neonates. We then evaluated the development of the gut and longissimus dorsi muscle in the newborn offspring. Additionally, antioxidative status, inflammatory responses, SCFA receptors, and the MAPK/NF-κB signaling pathway in both the intestine and longissimus dorsi muscle were examined. Correlation analyses between microbiota, oxidative stress, inflammation, and SCFA levels were also performed to explore the potential mechanisms underlying these effects.

## 2. Materials and Methods

### 2.1. Experimental Animals and Design

The experimental protocol received ethical approval from the Animal Care and Use Committee of the South China Agricultural University (SYXK2021-0231). This study was conducted in Guangdong Province, China. A total of 80 crossbred Landrace × Large White sows (parity 3–6) were selected based on parity, body condition score, and reproductive history, and were randomly divided into CON (basal diet) and GML (basal diet + 1000 mg/kg glyceryl monolaurate) groups (N = 40/group) in a randomized design. GML (CAS No. 142-18-7) with 95% purity was supplied by Hangzhou Kangyuan Food Technology Co. Ltd. (Hangzhou, Zhejiang, China). The basal diets met or exceeded NRC (2012) nutritional requirements for gestating sows. Throughout the experimental period, strict antibiotic-free protocols were maintained. Table S1 provides detailed information on the composition and nutritional levels of the basal diets.

### 2.2. Sample Gatherings

The experiment involved selecting Landrace × Large White sows (parity 3–6). From gestational day 85 until parturition, the sows stayed on their assigned experimental diets. During late gestation (day 85–107), the sows lived individually in gestation crates (2.1 × 0.9 m) with free access to water and received two feedings per day at 07:00 and 14:30, with a restricted amount of 3.0–3.5 kg per day. Seven days before farrowing, the sows moved to individual farrowing pens (2.1 × 1.6 m) maintained at 21–25 °C by automated ventilation. The farrowing process was monitored closely, and the team carried out regular cleaning protocols to ensure optimal hygiene while minimizing environmental disturbances.

On gestational day 110, a random selection of six healthy sows per group was made for sample collection. Concurrently, fresh fecal samples were obtained through rectal massage stimulation. During farrowing, one piglet per litter (selected based on proximity to average litter weight) from each of the six sampled sows per group was chosen for tissue collection. The sample size was determined based on efficacy analysis, aiming to detect significant differences while minimizing animal use. Sows and piglets were euthanized by intravenous injections in accordance with institutional guidelines. Blood samples of 5 mL were gathered from the anterior vena cava by applying techniques that minimized stress. Subsequently, the samples were centrifuged at 3500× *g* for 15 min at 4 °C. This process yielded plasma, which was then placed in storage at −80 °C. Dissected organs (heart, liver, spleen, kidneys, stomach, intestine, lungs) were weighed intact, with lung weights representing both left and right lobes. Tissues were rinsed in saline, flash-frozen in liquid nitrogen, or fixed in 4% paraformaldehyde for morphological analysis.

For intestinal morphology analysis, 1 cm segments of the jejunum from the midsection were fixed in 4% paraformaldehyde. Intestinal segments, each 2.5 cm in length, were longitudinally cut, rinsed with cold physiological saline, and immediately flash-frozen in liquid nitrogen for molecular analysis. At the thoracolumbar junction, dorsal muscle samples (1 cm × 1 cm × 2 cm) were collected and placed into cryogenic tubes.

### 2.3. Intestinal Morphology

Fix the tissue samples with formaldehyde. Gradually immerse the tissue in ethanol solutions of increasing concentrations to ensure effective dehydration. Wash with xylene and embed the tissue in paraffin to remove the ethanol and clean the surface. From each section, select six typical villi and their corresponding crypts. Measure the villus height (VH) and crypt depth (CD) in μm using ImageJ v1.8.0. Analyze six intact villi/crypts per sample from H&E-stained sections. Place the paraffin sections in gradient ethanol for 5 min, then treat them twice with dewaxing solution, each time for 10 min. Afterward, incubate the samples with the secondary antibody (FITC) at a dilution of 1:100 for 1 h at room temperature. Finally, observe the fluorescence using an Eclipse Ts2R-FL inverted microscope. For goblet cell staining, select 5 μm jejunal paraffin sections and stain them with a periodic acid-Schiff (PAS) staining kit (2312003, Solarbio, Beijing, China).

### 2.4. Analysis of Oxidative Stress Markers

An automatic low-temperature homogenizer (Sangon, Shanghai, China) was used to homogenize 250 mg of jejunum tissue samples in 0.45 mL of phosphate-buffered saline (PBS). The homogenate was then centrifuged at 4 °C for 10 min at 3500 rpm to obtain the supernatant. Levels of total antioxidant capacity (T-AOC), superoxide dismutase (SOD), glutathione (GSH), glutathione peroxidase (GSH-PX), and malondialdehyde (MDA) in the jejunum tissue and longissimus dorsi muscle supernatants were measured using commercial assay kits from Nanjing Jiancheng Bioengineering Institute, Nanjing, China. Details of the test kits are provided in Table S2.

### 2.5. RNA Extraction and qPCR Analysis

Fresh specimens of longissimus dorsi muscle and jejunum tissue were separately homogenized in lysis buffer (EZB-RN001, Roseville, MN, USA) with the help of a low-temperature homogenizer. Tissues (20–30 mg) were homogenized in 600 μL lysis buffer, while smaller samples (<20 mg) used 350 μL. When performing quantitative PCR, the reactions took place on an ABI PRISM 7500 SDS thermal cycler. In each reaction mixture, 2 μL of first-strand cDNA was added, along with 0.4 μL of the forward primer and 0.4 μL of the reverse primer, and the volume was adjusted to a total of 20 μL. Following a short centrifugation step, the samples underwent 40 cycles of amplification under the default fast cycling settings. The primer sequences employed in this research are presented in Table S3.

### 2.6. Western Blotting Analysis

To extract total protein from the jejunum and dorsal muscle, 0.6 mL of lysis buffer was carefully added to 70 mg of the respective tissue samples. Each sample had 30 μg of its total protein content extracted. This extracted protein fraction was then loaded onto a 10% SDS-PAGE gel for separation. Once the separation on the gel was finished, a transfer procedure was carried out to move the proteins onto a polyvinylidene difluoride (PVDF) membrane. Finally, the grayscale values of the bands were examined, and the relative abundance of the target protein was computed using image-processing software. Table S4 offers detailed information about the antibodies employed in this research. Note: each sample was used with an independent gel.

### 2.7. SCFA Analysis

Fecal samples (0.2 g) were processed in 1.5 mL tubes with 0.5 mL of 1 mmol/L 2-ethylbutyric acid solution, acidified to pH 2–3 using HCl, and incubated at 25 °C for 15 min with shaking. After centrifugation (10,000× *g*, 20 min), supernatants were analyzed via gas chromatography (GC-2014, Shimadzu, Kyoto, Japan) with nitrogen carrier gas (15 mL/min). The temperature program included: 100 °C (0.5 min), 180 °C at 8 °C/min (1 min), and 200 °C at 20 °C/min (15 min), with detector and inlet temperatures maintained at 250 °C and 230 °C, respectively.

### 2.8. The 16S rRNA Sequencing

Fresh fecal samples from the sows were collected and stored at −80 °C. Bacterial DNA was extracted using the MagPure Soil DNA LQ Kit (Magen, Guangdong, China). The V3-V4 region of the 16S rRNA gene was amplified with primers 341F (5′-CCTAYGGGGRBGCASCAG-3′) and 806R (5′-GGACTACNNGGGGTATCTAAT-3′). DNA concentration and purity were checked by 1% agarose gel electrophoresis. Qualified DNA samples were sequenced on an Illumina NovaSeq6000 to generate 250 bp paired-end reads. FLASH software 1.2.11 was used to merge the paired-end reads, and QIIME software (version 1.9.1) was applied for noise reduction and ASV feature extraction. Species annotation was performed to identify the microbial species. The sequencing and analysis were carried out by OE Biotechnology (Shanghai, China).

### 2.9. Statistical Assessment

In this study, the student’s *t*-test (SPSS 22.0) was used to assess significant differences in growth performance between the CON and GML groups, with the significance threshold set at *p* < 0.05. For 16S rRNA sequencing data, raw reads were quality-filtered and clustered into operational taxonomic units (OTUs) at 97% similarity using USEARCH v11. Taxonomic annotation was performed against the SILVA 138 database. Alpha diversity indices (Shannon, ACE, Chao1) and beta diversity (Bray–Curtis distance) were calculated using QIIME2. Independent sample *t*-tests and linear regression analyses were conducted for group comparisons and correlation assessments, respectively. Exact *p*-values are reported for all correlations. Significance thresholds were set at *p* < 0.1 (trend), 0.01 < *p* < 0.05 (significant), and *p* < 0.01 (highly significant). Results are expressed as mean ± SEM. KEGG pathway prediction and heatmap modeling were performed via the Cloud Tutu platform (https://tutucloudy.com/ accessed on 13 April 2024).

## 3. Results

### 3.1. Maternal GML Supplementation Enhanced Fetal Intrauterine Development

As depicted in Table 1, analyses of organ weight and organ index were conducted. Bowel weight, bowel index and lung index were significantly higher in the GML group than in the CON group (*p* < 0.05). Conversely, the indices for the heart, stomach, kidneys, and spleen revealed no statistically significant variations when comparing the two groups.

### 3.2. Maternal GML Supplementation Influenced Jejunum and Muscle Development in Newborn Piglets

**Figure 1:**
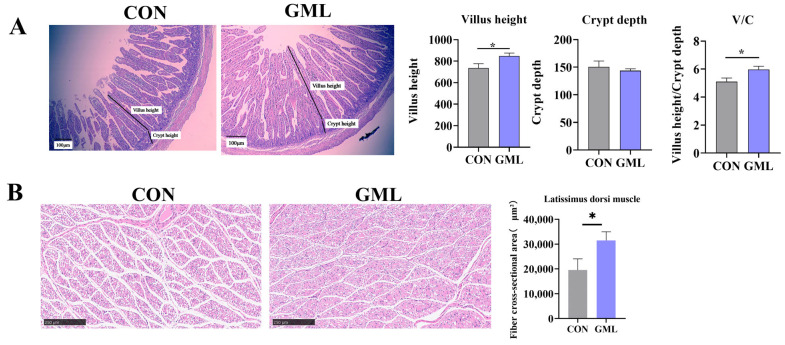
Placenta and longissimus dorsi muscle development. (**A**) CON and GML jejunal H&E staining; villus height, crypt depth, and the ratio of VH/CD in the CON and GML groups. (**B**) H&E staining of the longissimus dorsi muscle; data from newborn piglets (*n* = 6 per group), CON: control group; GML: glyceryl monolaurate group. * *p* < 0.05. (*n* = 6 per group). Differences were determined using *t*-tests (*p* < 0.05).

Intestinal and muscle development are critical for organismal growth. The intestine is crucial for nutrient digestion and absorption, with villus height and crypt depth being key indicators of its morphological integrity. Morphometric analysis revealed that GML supplementation enhanced jejunal architecture, demonstrating significant improvements in both villus height (*p* < 0.05) and the villus height-to-crypt depth ratio (*p* < 0.05) relative to control animals (Figure 1A). As shown in Figure 1B, GML treatment significantly increased muscle fiber cross-sectional area compared to CON (*p* < 0.05).

### 3.3. Maternal GML Supplementation Enhanced Intestinal Barrier Function in Neonatal Piglets

**Figure 2:**
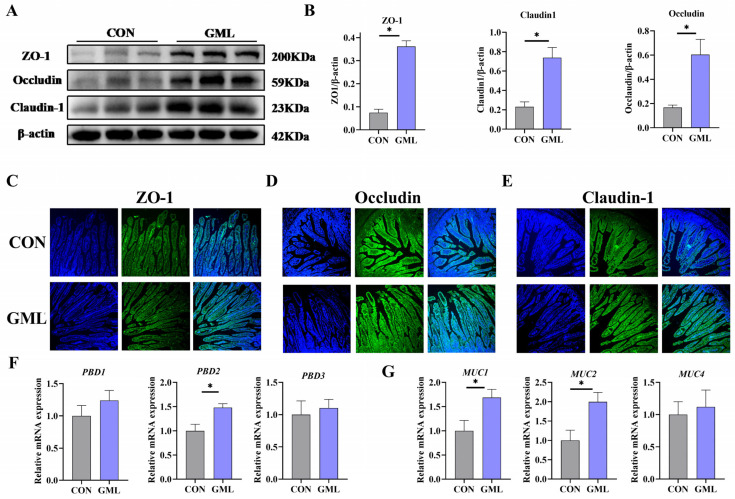
The development of intestinal barrier function in newborn piglets. (**A**,**B**) Relative abundance of tight junction proteins (ZO-1, occludin, and claudin-1) in the jejunum; (**C**–**F**) mRNA expression of β-defensins (*PBD1*, *PBD2*, *PBD3*); (**G**) mRNA expression of mucins (*MUC1*, *MUC2*, *MUC4*). * *p* < 0.05. Data from newborn piglets (*n* = 6 per group). Differences were determined using *t*-tests (*p* < 0.05).

Tight junctions are crucial for intestinal barrier integrity and mucosal permeability regulation, maintaining gut health [20]. Compared to CON, GML significantly increased ZO-1, occludin and claudin-1 protein expression (*p* < 0.05; Figure 2A,B). Immunofluorescence analysis of jejunal tissues demonstrated significantly enhanced staining intensities for tight junction proteins ZO-1, occludin, and claudin-1 in GML-treated samples versus controls (*p* < 0.05; Figure 2C–E).

The intestinal chemical barrier serves to prevent harmful substances and pathogens in the gut environment from entering the body. Intrauterine oxidative stress can compromise this barrier in piglets. The GML group showed significant upregulation of intestinal chemical barrier-related genes (*MUC1*, *MUC2*, *PBD2*; *p* < 0.05) compared to CON (Figure 2F,G).

### 3.4. Maternal GML Supplementation Promoted Longissimus Dorsi Muscle Development in Neonatal Piglets

**Figure 3:**
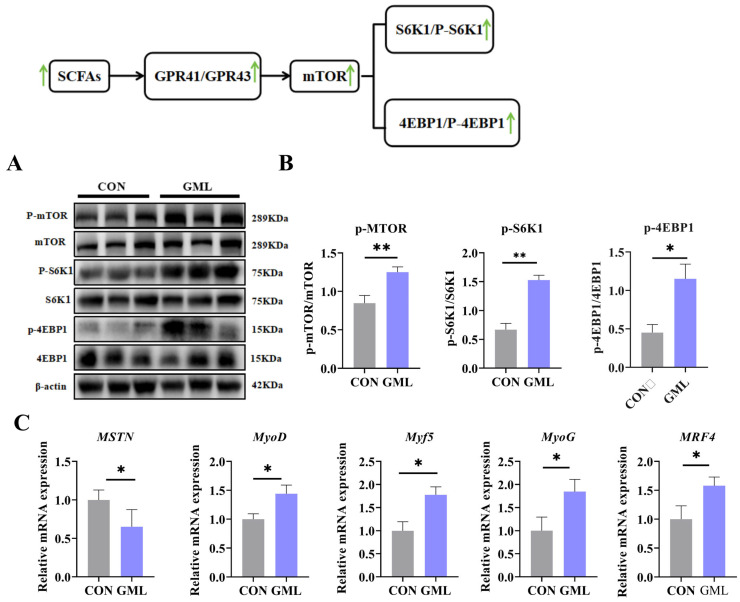
Development of the longissimus dorsi muscle in newborn piglets. (**A**,**B**) Effects of the GML group on the protein levels of mTOR, S6K1, and 4EBP1 in the longissimus dorsi muscle; (**C**) levels of muscle growth factors and growth inhibitors *MSTN*, *MyoD*, *Myf5*, *MyoG*, and *MRF4* in the longissimus dorsi muscle. * *p* < 0.05, ** *p* < 0.01. Data from newborn piglets (*n* = 6 per group). Differences were determined using *t*-tests (*p* < 0.05).

Muscle development is a critical determinant of overall growth potential [21]. As depicted in Figure 3A,B, the expression levels of P-mTOR, P-S6K1, and P-4EBP1 in the GML group were significantly higher than those in the CON group (*p* < 0.05). Furthermore, we investigated the expression profiles of key genes associated with muscle fiber growth in the longissimus dorsi muscle, encompassing *MSTN*, *MyoD*, *Myf5*, *MyoG*, and *MRF4*. The findings revealed that the GML group exhibited markedly elevated expression levels of *MyoD* (*p* < 0.05), *Myf5* (*p* < 0.05), *MyoG* (*p* < 0.05), and *MRF4* (*p* < 0.05) relative to the CON group, whereas *MSTN* expression was significantly downregulated (*p* < 0.05), as illustrated in Figure 3C.

### 3.5. Maternal GML Supplementation Altered the Intestinal Microbiota of Sows

**Figure 4:**
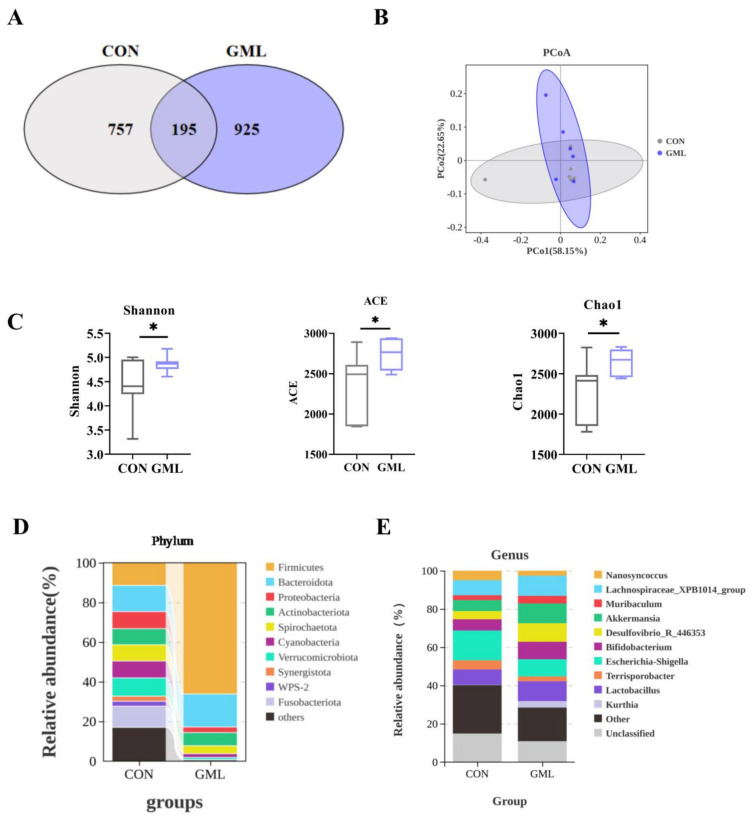
Microbial structure and diversity. (**A**) Venn diagram of OTUs; (**B**) principal coordinates analysis (pCoA) score plot; (**C**) Shannon index, ACE index, and Chao1 index of the microbial community; (**D**) relative abundance at the phylum level; (**E**) relative abundance at the genus level. CON: control group; GML: glyceryl monolaurate group. * *p* < 0.05. (*n* = 6 per group). Differences were determined using *t*-tests (*p* < 0.05).

The maternal gut microbiome undergoes dynamic changes during pregnancy, which are increasingly recognized as critical for fetal development [22]. Using 16S rRNA gene sequencing, we analyzed the fecal bacterial composition of pregnant sow at 110 days (Figure 4). Microbial analysis identified 925 and 757 unique OTUs in GML and CON groups, respectively, with 195 shared OTUs (Figure 4A). Both α-diversity indices (Shannon, ACE, Chao1) and β-diversity patterns demonstrated significant microbial community differences between groups (*p* < 0.05; Figure 4B,C).

**Figure 5:**
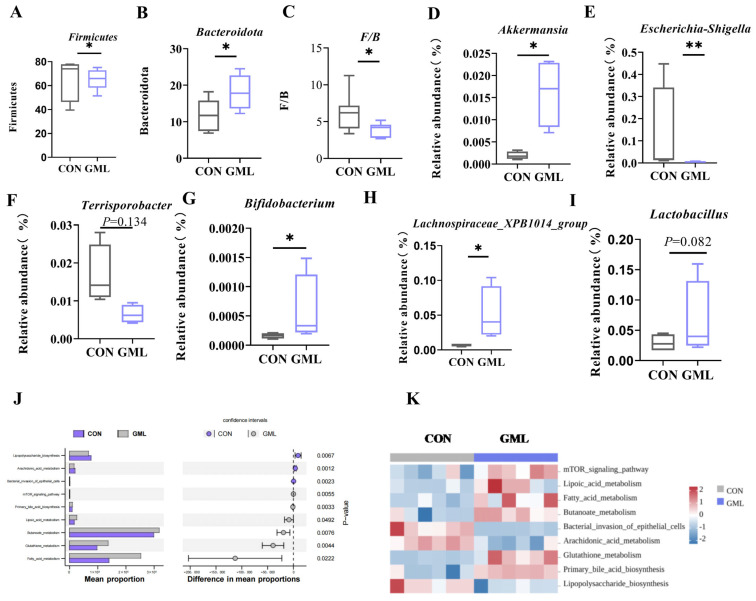
Analysis of gut microorganisms at the phylum and genus levels and KEGG functional prediction. (**A**–**I**) Analysis of microorganisms at the phylum and genus levels; microbial phylum and genus levels. (**J**,**K**) Differential prediction of KEGG metabolic pathways in fecal microbiota between the CON group and GML group. CON: control group; GML: glyceryl monolaurate group. KEGG: Kyoto Encyclopedia of Genes and Genomes. * *p* < 0.05; ** *p* < 0.01. (*n* = 6 per group). Differences were determined using *t*-tests (*p* < 0.05).

At the phylum level, the GML group had significant changes in Firmicutes abundance (Figure 5A) and a significant Bacteroidetes increase (*p* < 0.05; Figure 5B), with a lower Firmicutes-to-Bacteroidetes (F/B) ratio (*p* < 0.05; Figure 5C). At the genus level, dietary GML reduced *Escherichia-Shigella* abundance (*p* < 0.05; Figure 5E) and increased *Lactobacillus* (*p* < 0.05; Figure 5I) and *Akkermansia* (*p* < 0.05; Figure 5D). *Terrisporbacter* (*p* = 0.134), *Lachnospiraceae_XPB1014_group* (*p* < 0.05) and *Bifidobacterium* (*p* < 0.05) trended upward (Figure 5F–H).

The analysis of the KEGG pathways demonstrated that there were significant distinctions in the metabolic functions of the microbes between the two groups. GML supplementation notably influenced key metabolic pathways, including fatty acid metabolism, sulfur metabolism, glutathione metabolism, and lipopolysaccharide biosynthesis (Figure 5J,K).

### 3.6. Maternal GML Supplementation Increased SCFA Levels and Fatty Acid Receptor Expression

**Figure 6:**
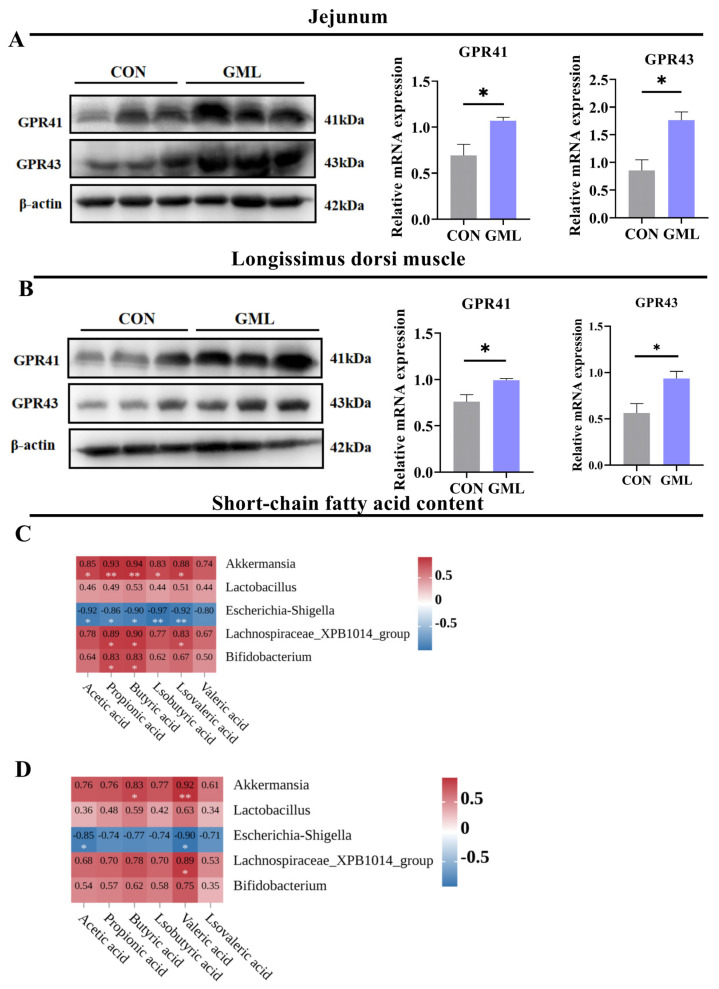
Short-chain fatty acid levels and fatty acid receptor expression. (**A**) Protein levels of fatty acid receptors GPR41 and GPR43 in the jejunum; (**B**) protein levels of fatty acid receptors GPR41 and GPR43 in the longest muscle of the back. (**C**,**D**) Levels of short-chain fatty acids in sow’s feces and plasma of piglets and their correlation with the major microorganisms. CON: control group; GML: glyceryl monolaurate group. * *p* < 0.05, ** *p* < 0.01. (*n* = 6 per group). Data were analyzed using Pearson correlation analysis; differences were determined using *t*-tests (*p* < 0.05).

SCFAs, which are the main metabolic byproducts of gut microbiota, are essential in regulating host metabolism, maintaining intestinal barrier integrity, supporting immune tolerance, and modulating autoimmune responses [23]. To evaluate the effects of maternal GML supplementation, we measured SCFA levels in sow feces and piglet plasma, as well as the expression of fatty acid receptors. Dietary GML supplementation significantly increased acetic acid and propionic butyric acid concentrations compared to the control group (*p* < 0.05; Table 2). Furthermore, compared with the control group, the GML group exhibited a significantly higher expression of fatty acid receptors GPR41 and GPR43 in both jejunal tissues and latissimus dorsi muscles (*p* < 0.05; Figure 6A,B). These results suggest that maternal GML supplementation modulates SCFA levels and enhances fatty acid receptor expression, promoting intestinal health and muscle development.

We further analyzed the relationship between SCFA levels and the abundance of intestinal microbial genera in sow feces (Figure 6C). Regression analysis demonstrated significant microbial–metabolite interactions, with *Akkermansia* abundance showing strong positive correlations with acetic acid (R^2^ = 0.85, *p* = 0.03), propionic acid (R^2^ = 0.93, *p* = 0.037), and isovaleric acid (R^2^ = 0.88, *p* = 0.02). Conversely, *Escherichia-Shigella* abundance exhibited pronounced inverse relationships with key short-chain fatty acids: acetic acid (R^2^ = 0.92, *p* = 0.032), propionic acid (R^2^ = 086, *p* = 0.042), and butyric acid (R^2^ = 0.90, *p* = 0.025). *Lachnospiraceae_XPB1014_group* abundance was positively correlated with propionic acid (R^2^ = 0. 89, *p* = 0.022) and butyric acid (R^2^ = 0.90, *p* = 0.032), while *Bifidobacterium* abundance showed positive correlations with propionic acid (R^2^ = 0.83, *p* = 0.043) and butyric acid (R^2^ = 0.83, *p* = 0.033). In piglet plasma, *Bifidobacterium* abundance was positively correlated with propionic acid (R^2^ = 0.57, *p* = 0.08), whereas acetic acid (R^2^ = 0.85, *p* = 0.024) and valeric acid (R^2^ = 0.90, *p* = 0.037) were negatively correlated with *Escherichia-Shigella* abundance (*p* < 0.05). Additionally, valeric acid (R^2^ = 0.89, *p* = 0.036) was positively correlated with *Lachnospiraceae_XPB1014_group* abundance (*p* < 0.05; Figure 6D).

### 3.7. Maternal GML Supplementation Reduced Oxidative Stress in Newborn Piglets

**Figure 7:**
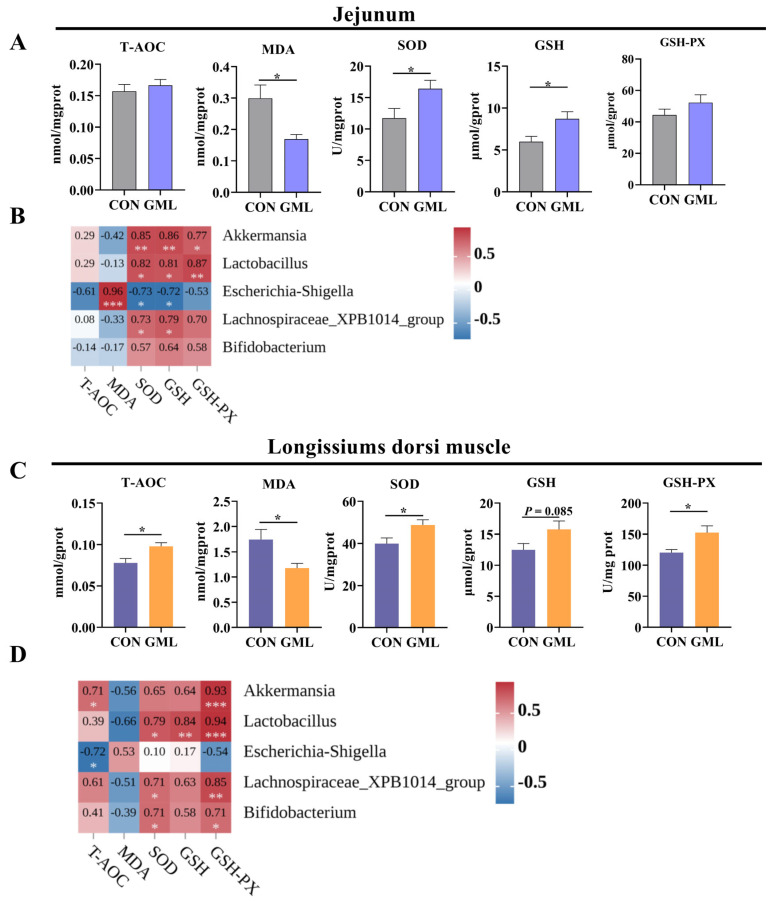
Oxidative stress levels in the jejunum and longissimus dorsi muscle of neonatal piglets. (**A**,**B**) T-AOC, MDA, SOD, GSH, and GSH-PX activities in the jejunum and their correlation with major microorganisms; (**C**,**D**) T-AOC, MDA, SOD, GSH, and GSH-PX activities in the longissimus dorsi muscle and their correlation with major microorganisms. CON: control group. GML: glyceryl monolaurate group. * *p* < 0.05, ** *p* < 0.01, ***p <= 0.001. Data from newborn piglets (*n* = 6 per group). Data were analyzed using Pearson correlation analysis; differences were determined using *t*-tests (*p* < 0.05).

Oxidative stress can significantly affect muscle and gut development [2]. Figure 7 illustrates the oxidative status of the jejunum. The analysis of jejunal oxidative stress markers showed that the GML group displayed significantly reduced MDA levels (*p* < 0.05), along with significantly elevated activities of SOD (*p* < 0.05) and GSH (*p* < 0.05) in contrast to the CON group. Additionally, GSH-PX (*p* < 0.05) and T-AOC (*p* < 0.05) showed an upward trend (Figure 7A). We further investigated the relationship between these oxidative stress markers and the abundance of gut microbial genera (Figure 7B). Regression analysis demonstrated significant positive correlations between *Akkermansia* abundance and antioxidant markers: GSH-PX (R^2^ = 0.77, *p* = 0.024), SOD (R^2^ = 0.85, *p* = 0.003), and GSH (R^2^ = 0.86, *p* = 0.005). Similar trends were observed for *Lactobacillus*, showing positive correlations with GSH-PX (R^2^ = 0.87, *p* = 0.009), SOD (R^2^ = 0.583, *p* = 0.017), and GSH (R^2^ = 0.81, *p* = 0.017). *Lachnospiraceae_XPB1014_group* abundance was also positively associated with GSH-PX (R^2^ = 0.70, *p* = 0.044), SOD (R^2^ = 0.73, *p* = 0.029), and GSH (R^2^ = 0.79, *p* = 0.018). In contrast, *Escherichia-Shigella* abundance had strong negative correlations with GSH-PX (R^2^ = 0.53, *p* = 0.028), SOD (R^2^ = 0.73, *p* = 0.031) and GSH (R^2^ = 0.72, *p* = 0.018).

In the longissimus dorsi muscle, oxidative stress markers were also assessed. Compared to the CON group, MDA levels in the GML group were significantly lower (*p* < 0.05), while the activities of SOD, GSH-PX, and T-AOC were significantly higher (*p* < 0.05). GSH levels also tended to increase (*p* < 0.05; Figure 7C). Regression analysis revealed distinct microbial–metabolic interactions between oxidative stress markers and gut microbiota in the longissimus dorsi muscle. Microbial–antioxidant correlations revealed distinct patterns: *Akkermansia* showed strong positive associations with GSH-PX (R^2^ = 0.93, *p* = 0.004), SOD (R^2^ = 0.65, *p* = 0.06), and GSH (R^2^ = 0.64, *p* = 0.0058), while *Lactobacillus* exhibited significant relationships with GSH-PX (R^2^ = 0.94, *p* = 0.003), SOD (R^2^ = 0.79, *p* = 0.022), and GSH (R^2^ = 0.84, *p* = 0.007). Conversely, *Escherichia-Shigella* displayed negative correlations with GSH-PX (R^2^ = 0.54, *p* = 0.051) and T-AOC (R^2^ = 0.72, *p* = 0.041). Positive associations were also observed for the *Lachnospiraceae_XPB1014_group* with GSH-PX (R^2^ = 0.85, *p* = 0.008) and GSH (R^2^ = 0.63, *p* = 0.058), and for *Bifidobacterium* with GSH (R^2^ = 0.58, *p* = 0.051) and SOD (R^2^ = 0.571, *p* = 0.048) (Figure 7D).

### 3.8. Maternal GML Supplementation Attenuated Inflammation in Neonatal Piglets

**Figure 8:**
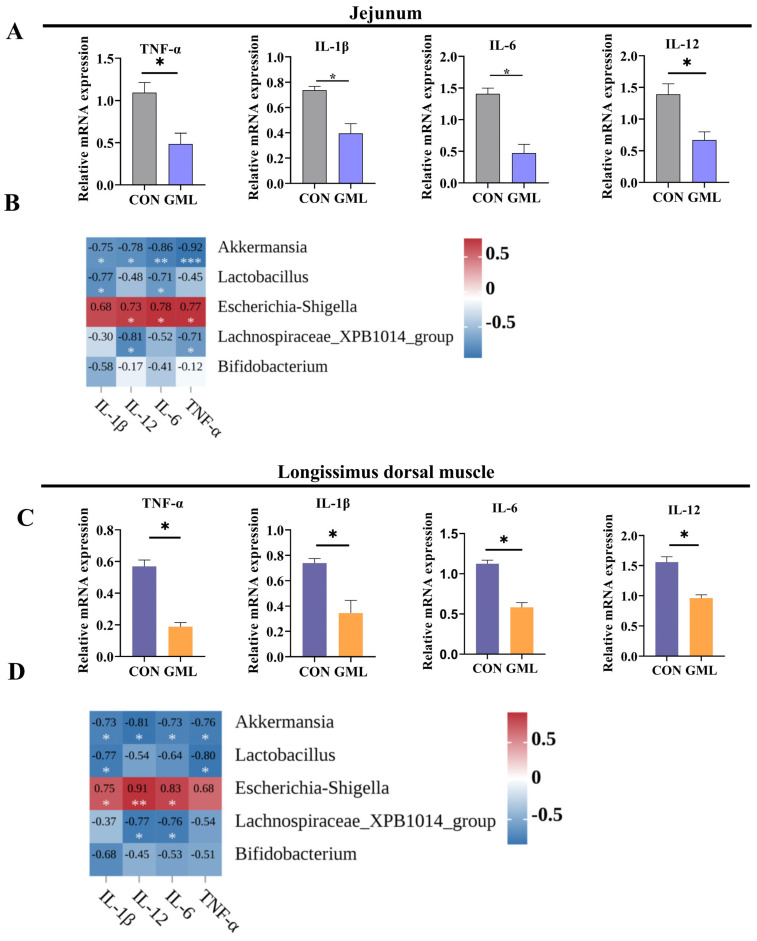
Inflammation levels in the jejunum and longissimus dorsi muscle of neonatal piglets (**A**,**B**) Levels of pro-inflammatory factors *TNF-α*, *IL-1β*, *IL-6*, and *IL-12* in the jejunum and their correlation with major microorganisms; (**C**,**D**) levels of inflammatory factors *TNF-α*, *IL-1β*, *IL-6*, and *IL-12* in the longissimus dorsi muscle and their correlation with major microorganisms. CON: control group; GML: glyceryl monolaurate group. * *p* < 0.05, ** *p* < 0.01; ***p ≤ 0.001. Data from newborn piglets (*n* = 6 per group). Data were analyzed using Pearson correlation analysis; differences were determined using *t*-tests (*p* < 0.05).

Oxidative stress is a key driver of inflammatory processes [24]; to investigate redox imbalance-induced inflammation, we analyzed pro-inflammatory gene expression (Figure 8A). The GML group showed significantly lower expressions of *IL-6*, *IL-1β*, *TNF-α*, and *IL-12* compared to CON (*p* < 0.05).

Microbial–inflammatory correlation analysis revealed distinct patterns (Figure 8B). *Lactobacillus* abundance negatively correlated with *IL-6* (R^2^ = 0.71, *p* = 0.02) and *IL-1β* (R^2^ = 0.77, *p* = 0.027), while *Escherichia-Shigella* showed positive correlations with *IL-6* (R^2^ = 0.78, *p* = 0.045), *IL-12* (R^2^ = 0.73, *p* = 0.039), *TNF-α* (R^2^ = 0.77, *p* = 0.021), and *IL-1β* (R^2^ = 0.68, *p* = 0.024). *Lachnospiraceae_XPB1014_group* abundance was negatively associated with *IL-12* (R^2^ = 0.81, *p* = 0.016) and *TNF-α* (R^2^ = 0.71, *p* = 0.049). *Akkermansia* displayed negative correlations with *IL-12* (R^2^ = 0.78, *p* = 0.044), *IL-1β* (R^2^ = 0.75, *p* = 0.023), *IL-6* (R^2^ = 0.86, *p* = 0.022), and *TNF-α* (R^2^ = 0.92, *p* = 0.003). In contrast, *Bifidobacterium* bifidum was negatively associated with *IL-12* (R^2^ = 0.17, *p* = 0.025) and *IL-1β* (R^2^ = 0.58, *p* = 0.042).

In the longissimus dorsi muscle, the GML group exhibited significantly reduced expressions of *IL-6*, *IL-1β*, *TNF-α*, and *IL-12* compared to CON (*p* < 0.05; Figure 8C). Regression analysis (Figure 8D) demonstrated negative correlations between specific gut microbiota and cytokine expression: *Lactobacillus* with *IL-1β* (R^2^ = 0.77, *p* = 0.026) and *TNF-α* (R^2^ = 0.80, *p* = 0.018); *Bifidobacterium* with *IL-12* (R^2^ = 0.45, *p* = 0.051), *IL-6* (R^2^ = 0.53, *p* = 0.053), and *IL-1β* (R^2^ = 0.68, *p* = 0.053). Similar negative associations were observed for *Lachnospiraceae_XPB1014_group* and *Akkermansia* with various cytokines.

### 3.9. Maternal GML Supplementation Suppressed MAPK/NF-κB Pathways

**Figure 9:**
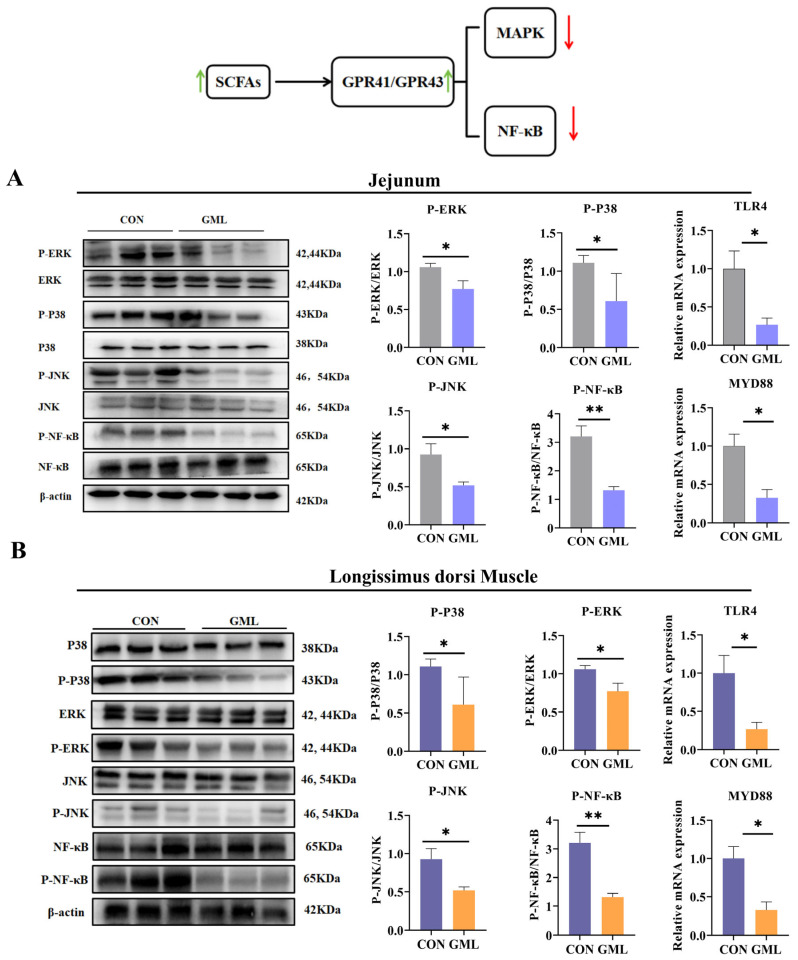
MAPK and NF-κB inflammatory signaling pathways in the jejunum and longissimus dorsi muscle of neonatal piglets. (**A**) Expression of MAPK (P-ERK/ERK, P-JNK/JNK, and P-P38/P38) and NF-κB signaling pathway-related proteins in the jejunum; (**B**) expression of MAPK (P-ERK/ERK, P-JNK/JNK, and P-P38/P38) and NF-κB signaling pathway-related proteins in the longissimus dorsi muscle. CON: control group; GML: glyceryl monolaurate group. * *p* < 0.05, ** *p* < 0.01. Red arrow: decrease. Green arrow: increase. Data from newborn piglets (*n* = 6 per group). Differences were determined using *t*-tests (*p* < 0.05).

Protein expression analysis revealed significant GML-mediated suppression of inflammatory pathways in both jejunal and muscular tissues. In the jejunum, the GML group demonstrated marked reductions in phosphorylated MAPK components (P-ERK, P-P38, P-JNK) and P-NF-κB, along with decreased *MYD88* and *TLR4* levels compared to CON (*p* < 0.05; Figure 9A). Parallel inhibition patterns were observed in the longissimus dorsi muscle, with significant downregulation of these signaling proteins and associated adaptor molecules (*p* < 0.05; Figure 9B).

## 4. Discussion

### 4.1. Maternal GML Supplementation Enhances Fetal Development and Growth

It has been well demonstrated that the inclusion of antioxidants and bioactive compounds such as selenium yeast and resveratrol in the maternal diet is vital for optimal fetal development [25,26]. Notably, our data suggest that maternal GML supplementation positively influences fetal development, as evidenced by increased intestinal weight, organ indices, and lung development in piglets, highlighting the beneficial impact of GML on neonatal growth. This explains GML’s ability to promote SCFA-producing bacteria like Lactobacillus and *Akkermansia,* while also inhibiting harmful bacteria like *Escherichia and Shigella* [27]. Research emphasizes the crucial role of the digestive system in fetal growth and development [28]. Stressful conditions, for example, were shown to alter the structural integrity and physiological functions of the small intestine in neonatal piglets leading to diminished digestive and absorptive efficiency, as well as compromised intestinal barrier integrity [29]. These adverse effects often manifest as reduced feed consumption, elevated incidence of diarrhea, and impaired growth performance [30]. Muscle development is also vital for fetal growth; such as intrauterine growth restriction (IUGR), which may result in a decreased number of muscle fibers at birth [31]. Our research revealed that dietary supplementation with GML led to a notable increase in muscle fiber numbers. Additionally, stress in neonatal piglets has been associated with structural damage to the villus-crypt architecture [32,33]. Our research shows that maternal GML supplementation enhanced the jejunal architecture in offspring, demonstrating significant increases in both absolute villus height and the VH/CD ratio compared to control groups (*p* < 0.05). These findings collectively indicate that incorporating GML into the maternal diet during gestation supports the development of both intestinal and muscular tissues in the progeny. Consistent with its predicted metabolic effects (Introduction), GML elevated SCFA-producing bacteria (Figure 5D,I), explaining enhanced intestinal weight (Table 1) and muscle fiber development (Figure 1B).

### 4.2. Impact of Maternal GML on Intestinal Barrier and Muscle Development

A robust and functional intestinal barrier is vital for the efficient absorption of essential nutrients required for rapid growth, serving as a foundation for overall developmental processes [34]. This barrier consists of biochemical components (defensins, antimicrobial peptides, mucins) and structural tight junction proteins (ZO-1, occludin, claudin-1) [35]. These components collectively ensure gut integrity and health by preventing the infiltration of harmful agents, regulating immune homeostasis, and reducing inflammation and pathogen invasion [36]. Maternal dietary enrichment with GML during gestation led to a significant upregulation of *PBD2*, *MUC1*, and *MUC2* gene expression. Concurrently, the offspring’s intestinal tissues displayed elevated levels of ZO-1, occludin, and claudin-1 proteins. Earlier research underscores the strong association between mTOR pathway activation in muscle and muscle growth enhancement [37]. Some studies show that GML supplementation promotes fetal intestinal development [25]. In our experiments, maternal GML intake led to a significant upregulation of P-mTOR, P-S6K1, and P-4EBP1 protein expression in the jejunum of offspring. This was accompanied by increased expression of key muscle fiber growth genes, such as *MyoD*, *Myf5*, *MyoG*, and *MRF4*. The increased expression of these genes highlights the crucial role of GML in promoting the development of both intestinal and muscular tissues.

### 4.3. Gut Microbiota Modulation and Its Role in Maternal–Fetal Health

Maternal microbial metabolites can translocate into fetal circulation via placental transfer, directly modulating developmental processes [6]. Research has found that systemic LPS translocation from mother to fetus can be alleviated by reshaping the composition of the gut microbiota. This also enhances the antioxidant status of the placenta and fetus during pregnancy [25]. The study reveals that intestinal microbial imbalances are linked to diverse pathologies, including gestational disorders and compromised pregnancy outcomes [38,39]. Significant differences in microbial communities were found between CON and GML groups, the F/B ratio decreased while the presence of beneficial bacteria like *Lactobacillus*, *Lachnospiraceae_XPB1014_group*, and *Akkermansia* increased. KEGG analysis revealed significant enrichment in metabolic pathways, including fatty acid metabolism, lipoate metabolism, glutathione metabolism, and LPS biosynthesis. These pathways are all associated with antioxidative processes. As anticipated, SCFAs mediate gut–placenta crosstalk (Introduction), as evidenced by correlations between *Akkermansia* and fecal acetate (R^2^ = 0.85, *p* = 0.03; Figure 6C) alongside upregulated GPR41/43 (Figure 6A,B). To determine whether the changes in SCFA levels following GML supplementation were linked to the intestinal microbiota, we performed correlation analyses between microbial populations and SCFA concentrations. SCFA levels are significantly positively correlated with the abundance of *Lactobacillus*, *Lachnospiraceae_XPB1014_group*, *Akkermansia*, and *Bifidobacterium*. Conversely, SCFA levels are significantly negatively correlated with *Escherichia-Shigella* abundance. Specifically, GML supplementation increases beneficial SCFA-producing bacteria such as *Akkermansia*, while it inhibits pathogenic taxa such as *Escherichia-Shigella*, thereby remodeling the microbial structure and promoting SCFA biosynthesis. These results point to the fact that maternal GML supplementation effectively modulates fecal SCFA concentrations and upregulates fatty acid receptor expression, potentially improving gut health and metabolic regulation.

### 4.4. Anti-Inflammatory and Antioxidant Effects of Maternal GML Supplementation

The results of our study showed that GML administration significantly enhanced T-AOC, SOD, GSH, and GSH-PX levels while reducing MDA levels in the jejunum and longissimus dorsi muscle. These results indicate that GML effectively mitigates oxidative stress in intestinal and muscular tissues. Furthermore, we also found gestational GML supplementation significantly suppressed pro-inflammatory cytokine expression and inhibited *TLR4*/*MYD88* signaling mediators in both the jejunum and longissimus dorsi of neonatal piglets, which are consistent with the theory of supporting the inflammation–development axis proposed earlier, We found that maternal GML supplementation effectively lowers the expression of P-ERK, P-JNK, P-P38, and P-NFκB proteins in the intestines and muscles of offspring. To explore whether the anti-inflammatory and antioxidant effects of GML are mediated by the gut microbiota, we conducted correlation analyses between microbial populations and these capacities. Oxidative stress levels in the jejunum and longissimus dorsi of neonatal piglets were found to correlate positively with the abundance of *Lactobacillus*, *Lachnospiraceae_XPB1014_group*, *Akkermansia*, and *Bifidobacterium*. However, they were negatively correlated with *Escherichia-Shigella* abundance. Inflammation levels in the same tissues were inversely associated with the abundance of *Lactobacillus*, *Lachnospiraceae_XPB1014_group*, *Akkermansia*, and *Bifidobacterium*, while positively associated with *Escherichia-Shigella* abundance. The results indicated that the enhanced anti-inflammatory and antioxidant capacities induced by GML supplementation are closely linked to gut microbiota modulation. The MAPK and NF-κB signaling pathways are well-known pro-inflammatory regulators of stress responses and apoptosis [40,41]. Our study indicates that maternal GML supplementation suppresses the MAPK/NF-κB inflammatory pathway, reveals its role in ameliorating inflammatory and oxidative conditions in the offspring’s gut and longissimus dorsi muscle, as well as supporting the development of newborn piglets. Our research findings can help the production industry by reducing piglet mortality and reducing the use of antibiotics. However, this study has the following limitations. First, only a single dose of GML (1000 mg/kg) was tested. Although this dose significantly promoted fetal development (Table 1, Figure 1) and microbiota regulation (Figure 5), future studies should [40,41] explore dose-dependent effects (e.g., 500, 1000, 1500 mg/kg) to determine the optimal dose level for maternal and fetal outcomes. Second, although the pig model has physiological relevance due to its similarity to human placental structure and intestinal development [42,43], direct extrapolation of results to human pregnancy should be approached with caution. Furthermore, related studies have shown that lauric acid may improve skin health by reducing skin inflammation and inhibiting bacterial growth [44]. The effects of GML on human pregnancy-related microbiota and fetal antioxidant capacity have not been validated. Despite these limitations, our findings establish a mechanistic framework for GML as a microbiota-targeted intervention to alleviate fetal oxidative stress, with implications for livestock health and human intrauterine growth restriction (IUGR) research.

## 5. Conclusions

Maternal GML supplementation increases SCFA levels by increasing the abundance of SCFA-producing bacteria (*Lactobacillus* and *Akkermansia*) and inhibiting pathogens (*Escherichia-Shigella*) in the sow. These SCFAs enhanced intestinal barrier function (through GPR41/GPR43-mediated upregulation of tight junctions) and muscle development (through *MyoD*/*MRF4* and mTOR activation). Meanwhile, GML supplementation enhanced antioxidant and anti-inflammatory effects (inhibition of MAPK/NF-κB), attenuated intrauterine oxidative stress and promoted fetal growth. This study emphasizes the dual role of GML in regulating the gut microbiota and short-chain fatty acid metabolism to promote offspring development.

## Figures and Tables

**Table 1:** Effect of supplementation with glyceryl monolaurate on organ weights and indices.

Item	CON	GML	SEM	*p*-Value
Organ index (%)				
*n*	6	6		
Heart	0.008	0.008	0.00021	0.971
Liver	0.029	0.028	0.00069	0.660
Spleen	0.001	0.001	0.00011	0.531
Kidney	0.009	0.010	0.00032	0.122
Stomach	0.016	0.012	0.00187	0.244
Intestine	0.054	0.002	0.00262	0.001
Lung	0.017	0.030	0.00245	0.002
Organ weight (g)				
*n*	6	6		
Heart	14.65	12.55	0.75	0.172
Liver	53.09	44.36	3.14	0.176
Spleen	2.53	2.51	0.25	0.968
Kidney	17.22	16.15	0.88	0.565
Stomach	29.80	18.15	3.29	0.074
Intestine	95.60	126.95	5.95	0.002
Lung	30.88	33.69	1.98	0.506
Body weight	1830.6	1548.95	0.21	0.089

All values are expressed as means ± SEM. SEM = pooled standard error of the mean. Differences were statistically significant when *p* < 0.05. Organ index (%) = organ weight/animal weight. CON: control group, sows fed basal diet; GML: glycerol monolaurate group, sows fed basal diet supplemented with 1000 mg/kg GML.

**Table 2:** SCFA levels in sow feces and newborn piglet plasma.

Item	CON	GML	SEM	*p*-Value
N	6	6		
Feces, μmol/g				
Acetic acid	15.73	36.19	2.823	0.0069
Propionic acid	4.80	8.43	1.323	0.0443
Butyric acid	1.83	4.78	0.618	0.1144
Isobutyric acid	2.77	2.80	0.107	0.8945
Valeric acid	1.37	2.59	0.057	0.0043
Isovaleric acid	1.45	5.86	0.886	0.0144
Plasma, μmol/g				
Acetic acid	41.80	54.41	0.683	0.0002
Propionic acid	3.24	6.17	0.247	0.0013
Butyric acid	1.38	2.05	0.055	0.0017
Isobutyric acid	0.93	1.31	0.017	0.0001
Valeric acid	1.32	1.51	0.044	0.0428
Isovaleric acid	1.56	2.45	0.064	0.0007

All values are expressed as means ± SEM. SEM = pooled standard error of the mean. Differences were statistically significant when *p* < 0.05. CON: control group, sows fed basal diet; GML: glycerol monolaurate group, sows fed basal diet supplemented with 1000 mg/kg GML.

## Data Availability

The original contributions generated for this study are included in the article, further inquiries can be directed to the corresponding author. Informed consent was obtained from all subjects involved in the study.

## Supplementary Materials

The following supporting information can be downloaded at: https://www.mdpi.com/article/10.3390/antiox14070783/s1, Table S1: Ingredient composition and nutritional levels of the basic diet (air-dried basis, %); Table S2: Reagent kit information related to chemical analysis; Table S3: Primer sequences used in real-time PCR; Table S4: Information related to primary and secondary antibodies in the Wenstern blot.

## Abbreviations

CON: control; GML: glycerol monolaurate; PCoA: principal coordinate analysis; T-AOC: total antioxidant capacity; MDA: malondialdehyde; SOD: superoxide dismutase; GSH: glutathione; GSH-PX: glutathione peroxidase; *TNF-α*: tumor necrosis factor-α; *IL-1β*: interleukin-1β; *IL-6*: interleukin-6; *IL-12*: interleukin-12; *TLR4*: oll-like receptor 4; *MYD88*: myeloid differentiation factor 88; P38: P38 MAPK; P-P38: phospho-p38 MAPK; JNK: C-Jun N-terminal protein kinase; P-JNK: phospho-c-Jun N-terminal protein kinase; ERK: extracellular signal-regulated kinase; P-ERK: phospho-extracellular signal-regulated kinase; NF-κb: nuclear factor kappa-B; P-NF-κB: phospho-nuclear factor kappa-B; ZO-1: zonula occludens protein 1; *PBD1*: porcine β-defensin-1; *PBD2*: porcine β-defensin-2; *PBD3*: porcine β-defensin-3; *MUC1*: mucin 1; *MUC2*: mucin 2; *MUC4*: mucin 4; P-MTOR: phospho-mammalian target of rapamycin; P-S6K1: phospho-ribosomal protein S6 kinase beta-1; P-4EBP1: phospho-4E-binding protein1; *MSTN*: myostatin; *MyoD*: myoblast determination protein; *Myf5*: myogenic factor 5; *MyoG*: myogenin; *MRF4*: myogenic regulatory factor 4; ACE: abundance-based coverage estimator; GPR41: G-protein-coupled receptor 41; GPR43: G-protein-coupled receptor 43; SCFAs: short-chain fatty acids; LPS: lipopolysaccharides.
