# Supplementary material for: Dietary Glyceryl Monolaurate Supplementation During Pregnancy Enhances Fetal Intrauterine Development and Antioxidant Capacity in Sows via Microbiota Modulation
Source: Antioxidants (Basel). 2025 Jun 25;14(7):783. doi: 10.3390/antiox14070783 (PMC12291777; doi:10.3390/antiox14070783)
Supplement: Supplementary file 1 [file antioxidants-14-00783-s001.zip › antioxidants-3686118-supplementary.pdf]

# Supporting Information

**Table S1.** Ingredient composition and nutritional levels of the basic diet (air-dried basis, %)

| Item | Ingredients (%) |
|---|---|
| Corn | 21.60 |
| Soybean meal | 15.80 |
| Wheat | 18.00 |
| Barley | 24.80 |
| Soybean shee | 14.00 |
| Fish meal | 0.60 |
| Soybean oil | 0.50 |
| Dicalcium phosphate | 1.80 |
| Limestone | 0.68 |
| Salt | 0.40 |
| Choline chloride (50%) | 0.16 |
| Pregnant vitamin and mineral premix 1 | 1.56 |
| Mold inhibitor | 0.10 |
| Total | 100.00 |
| Nutrient level 2 | |
| Digestible energy, MJ/kg | 11.56 |
| Crude protein | 12.8 |
| Crude fiber | 9.36 |
| Crude fat | 4.36 |
| Ash | 5.32 |
| Calcium | 0.70 |
| Phosphorus | 0.60 |
| Available phosphorus | 0.53 |
| SID Lys | 0.77 |
| SID Met + Cys | 0.51 |
| SID Thr | 0.49 |
| SID Trp | 0.15 |

[1] Pregnant vitamin and mineral premix provide per kg of complete diet: 5000 IU vitamin A, 1500 IU vitamin $D_3$, 50 IU vitamin E, 3 mg vitamin $K_3$, 3 mg vitamin $B_1$, 7.5 mg vitamin $B_2$, 4.4 mg vitamin B6, 0.024 mg vitamin B12, 0.11 mg I ($CaI_2O_6$), 0.20 mg selenium, 35 mg niacin, 30 mg D-pantothenate, 3.0 mg folic acid, 0.14 mg D-biotin, 75 mg Zn ($ZnSO_4 \cdot H_2O$), 95 mg Fe($FeSO_4 \cdot H_2O$), 37 mg Mn ($MnSO_4 \cdot H_2O$), 17 mg Cu ($CuSO_4 \cdot 5H_2O$).

[2] Digestible energies standardized ileal digestibility (SID) amino acids and available phosphorus are calculated values, and all other nutritional levels are measured values.

**Table S2.** Reagent kit information related to chemical analysis.

| Kits | Product name | Code No. | Company |
|---|---|---|---|
| T-AOC | Total antioxidant capacity assay kit (ABTS method) | A-015-2-1 | Nanjing JianCheng Bioengineering Institute |
| T-SOD | (Hydroxylamine method) Total Superoxide Dismutase (T-SOD) assay kit | A001-1-2 | Nanjing JianCheng Bioengineering Institute |
| GSH-Px | Glutathione peroxidase (GSH-PX) assay kit | A005-1-2 | Nanjing JianCheng Bioengineering Institute |
| GSH | Reduced glutathione (GSH) assay kit | A006-2-1 | Nanjing JianCheng Bioengineering Institute |
| MDA | Malondialdehyde (MDA) assay kit (TBA method) | A003-1-2 | Nanjing JianCheng Bioengineering Institute |
| Lysis buffer | | EZB-RN001 | EZBioscience,USA |
| Tissue RNA Purification | | EZB-RN001 | EZBioscience,USA |
| Colour Reverse Transcription Kit (with gDNA Remover) | | A0010CGQ | EZBioscience,USA |
| Colour SYBR Green qPCR Master Mix kit | | A0012-R2 | EZBioscience,USA |
| RIPA Lysis Buffer P0013B Beyotime | | P0013B | Beyotime, Shanghai, China |
| BLOT-QuickBlocker | | C006011 | Sangon, Shanghai,China |
| BCA Protein Assay Kit | | P0012 | Beyotime, Shanghai, China |
| ECL Plus chemiluminescence detection kit | | P1010-250 | Applygen Technologies Inc, Beijing, China |
| Environmentally friendly dewaxing transparent liquid | | G1128-1L | Servicebio |

| | | |
|---|---|---|
| 20 × Tris EDTA antigen repair solution (pH 8.0) | G1206-250ML | Servicebio |
| Bovine serum albumin BSA | GC305010-25g | Servicebio |
| DAPI staining reagent | G1012-10ML | Servicebio |
| Periodic Acid Schiff (PAS) Stain Kit | 2312003 | Solarbio |

**Table S3.** Primer sequences used in real-time PCR.

| Gene Name | Position | Primer Sequences (5'→3') |
|---|---|---|
| *TNF-α* | Forward | CCACCAACGTTTTCCTCACT |
| | Reverse | TAGTCGGGCAGGTTGATCTC |
| *IL-1β* | Forward | TCTGCCCTGTACCCCAACTG |
| | Reverse | CCAGGAAGACGGGCTTTTG |
| *IL-6* | Forward | TGGCTACTGCCTTCCCTACC |
| | Reverse | AGAGCCTGCATCAGCTCAGT |
| *IL-12* | Forward | CAACCCTGTGCCTTAGCAGT |
| | Reverse | AGAGCCTGCATCAGCTCAGT |
| *PBD1* | Forward | TTCTTCGGGCTGTTGCTACT |
| | Reverse | AGGTGCCGATCTGTTTCATCTTTGG |
| *PBD2* | Forward | ATTAACCTGCTTACGGGTCTTGGC |
| | Reverse | CCACTGTAACAGGTCCCTTCAATCC |
| *PBD3* | Forward | TCTTCTTGTTCCTGATGCCTCTTCC |
| | Reverse | GCCACTCACAGAACAGCTACCTATC |
| *MUC1* | Forward | TTCTTCGGGCTGTTGCTACT |
| | Reverse | ACTGTCTTGGAAGGCCAGAA |
| *MUC2* | Forward | CTCCATCCGCTTCAGAAAAG |
| | Reverse | ACTACCTGGGGCCTCTGAAT |
| *MUC4* | Forward | CCTCCCAAGCAGATGTCAAT |
| | Reverse | CTGGGATAAGAATGCCTCCA |
| *β-actin* | Forward | GATCTGGCACCACACCTTCTACAAC |
| | Reverse | TCATCTTCTCACGGTTGGCTTTGG |
| *Myf5* | Forward | GATCAGCAACTCCGAGCAACC |
| | Reverse | ATGGTAGATGAGCCTGGAACTAGAC |
| *MyoD* | Forward | CGCAACGCCATCCGCTATATC |
| | Reverse | AGTCACCGCTGTAGTGCTCTC |
| *MRF4* | Forward | CCTCAGCCTCCAGCAGTCTTC |
| | Reverse | TACTTCTCCACCACCTCCTCCAC |
| *Myog* | Forward | CGCAGGCTCAAGAAGGTGAATG |
| | Reverse | AGGCACTCGATGTACTGGATGG |
| *MSTN* | Forward | AGACCCGTCAAGACTCCTACAAC |
| | Reverse | CTTCACATCAATGCTCTGCCAAATAC |

**Table S4.** Information related to primary and secondary antibodies in the Wenstern blot.

| Protein | Product name | Code No. | Company |
|---|---|---|---|
| P38 | p38 MAPK Monoclonal antibody | 66234-1-Ig | Proteintech |
| P-P38 | Phospho-p38 MAPK (Thr180/Tyr182) (3D7) Rabbit mAb (Biotinylated) 4092 | 4092S | Cellsignaling |
| JNK | SAPK/JNK Antibody #9252 | 9252S | Cellsignaling |
| P-JNK | Phospho-SAPK/JNK (Thr183/Tyr185) (81E11) Rabbit mAb #4668 | 4668S | Cellsignaling |
| ERK | p44/42 MAPK (Erk1/2) Antibody #9102 | 9102S | Cellsignaling |
| P-ERK | Phospho-p44/42 MAPK (Erk1/2) (Thr202/Tyr204) Antibody #9101 | 9101S | Cellsignaling |
| NF-κB | NF-κB p65 Polyclonal antibody | 10745-1-AP | Proteintech |
| P-NF-κB | Phospho-NF-κB p65 (Ser536) (93H1) Rabbit mAb #3033 | 3033S | Cellsignaling |
| ZO-1 | ZO-1 Polyclonal antibody | 21773-1-AP | Proteintech |
| mTOR | mTOR antibody | ab2732 | Cellsignaling |
| P-mTOR | Phospho-mTOR (Ser2448) Antibody | 2971 | Cellsignaling |
| S6K | p70 S6 Kinase Antibody | 9202 | Cellsignaling |
| P-S6K | Phospho-p70 S6 Kinase (Thr389) Antibody | 9205 | Cellsignaling |
| 4EBP1 | 4E-BP1 Antibody | ab32024 | Cellsignaling |
| P-4EBP1 | Phospho-4E-BP1 (Thr37/46) (236B4) Rabbit mAb | 2855 | Cellsignaling |
| β-actin | β-Actin Antibody | Ab8226 | Cellsignaling |
| Occludin | Occludin Polyclonal antibody | 27260-1-AP | Proteintech |
| Claudin-1 | Claudin-1 Polyclonal antibody | 13050-1-AP | Proteintech |
| secondary antibody | Goat Anti-Rabbit IgG H&L | 511203 | Zenbio |
| secondary antibody | Goat Anti-Mouse IgG H&L | 511103 | Zenbio |
| secondary antibody | FITC-labeled goat anti-rabbit IgG | GB22303 | Servicebio |
